# The Usability and Impact of a Low-Cost Pet Robot for Older Adults and People With Dementia: Qualitative Content Analysis of User Experiences and Perceptions on Consumer Websites

**DOI:** 10.2196/29224

**Published:** 2022-02-22

**Authors:** Wei Qi Koh, Sally Whelan, Pascale Heins, Dympna Casey, Elaine Toomey, Rose-Marie Dröes

**Affiliations:** 1 School of Nursing and Midwifery College of Medicine, Nursing & Health Sciences National University of Ireland Galway Ireland; 2 Irish Centre for Autism & Neurodevelopmental Research National University of Ireland Galway Galway Ireland; 3 Department of Psychiatry and Neuropsychology School for Mental Health and Neuroscience Maastricht University Maastricht Netherlands; 4 School of Allied Health University of Limerick Limerick Ireland; 5 Department of Psychiatry Amsterdam University Medical Centers VUmc/Amsterdam Public Health Research Institute Amsterdam Netherlands

**Keywords:** social robot, pet robots, low-cost robot, dementia, older adults, qualitative research, qualitative content analysis

## Abstract

**Background:**

Worldwide, populations are aging exponentially. Older adults and people with dementia are especially at risk of social isolation and loneliness. Social robots, including robotic pets, have had positive impacts on older adults and people with dementia by providing companionship, improving mood, reducing agitation, and facilitating social interaction. Nevertheless, the issue of affordability can hinder technology access. The Joy for All (JfA) robotic pets have showed promise as examples of low-cost alternatives. However, there has been no research that investigated the usability and impact of such low-cost robotic pets based on perceptions and experiences of its use with older adults and people with dementia.

**Objective:**

The aim of our study was to explore the usability and impact of the JfA robotic cat, as an example of a low-cost robot, based on perceptions and experiences of using the JfA cat for older adults and people with dementia.

**Methods:**

We used a novel methodology of analyzing a large volume of information that was uploaded by reviewers of the JfA cat onto online consumer review sites. Data were collected from 15 consumer websites. This provided a total of 2445 reviews. Next, all reviews were screened. A total of 1327 reviews that contained information about use of the JfA cat for older adults or people with dementia were included for analysis. These were reviews that contained terms relating to “older adults,” “dementia,” and “institutional care” and were published in the English language. Descriptive statistics was used to characterize available demographic information, and textual data were qualitatively analyzed using inductive content analysis.

**Results:**

Most reviews were derived from consumer sites in the United States, and most reviewers were family members of users (ie, older adults and people with dementia). Based on the qualitative content analysis, 5 key themes were generated: prior expectations, perceptions, meaningful activities, impacts, and practicalities. Reviewers had prior expectations of the JfA cat, which included circumstantial reasons that prompted them to purchase this technology. Their perceptions evolved after using the technology, where most reported positive perceptions about their appearance and interactivity. The use of the robot provided opportunities for users to care for it and incorporate it into their routine. Finally, reviewers also shared information about the impacts of device and practicalities related to its use.

**Conclusions:**

This study provides useful knowledge about the usability and impact of a low-cost pet robot, based on experiences and perceptions of its use. These findings can help researchers, robot developers, and clinicians understand the viability of using low-cost robotic pets to benefit older adults and people with dementia. Future research should consider evaluating design preferences for robotic pets, and compare the effects of low-cost robotic pets with other more technologically advanced robotic pets.

## Introduction

Worldwide, the population is aging exponentially. Since the prevalence of dementia greatly increases with age, the corresponding number of people with dementia is also on the rise [1]. Older adults and people with dementia are especially at risk of social isolation and reduced psychosocial health [2]. Social robots, such as robotic pets, are innovative technological solutions that are being developed and deployed to address the psychosocial needs of this population [3]. They are defined as autonomous or semiautonomous devices that are socially evocative and socially receptive [4], with the ability to interact with humans in a socially appropriate manner [5]. Pet robots are developed to simulate and substitute animal-assisted therapy [6]. Although animal-assisted therapy can benefit the social and emotional health of older adults and people with dementia by providing companionship, eliciting relaxation, and reducing loneliness [7,8], the use of live animals can pose several challenges. For instance, there is potential for transmission of zoonotic diseases, animal aggression, and compromised animal welfare [9]. Therefore, the use of a robotic alternative is seen as a novel way to enable older people and people with dementia to reap the psychosocial benefits of animal-assisted therapy, while potential adverse effects are avoided. Overall investigations into their effects have demonstrated positive benefits for older adults and people with dementia. Their use was found to have positively affected physiological indicators through improved sleep, improved oxygenation and cardiac status, reduced use of psychotropic drugs, improved mood, and improved social engagement [10-12]. PARO, a robotic seal, was the most studied robotic pet. Other pet robots include AIBO (robotic dog), JustoCat and NeCoRo cat (robotic cats), and Pleo (robotic dinosaur). However, the affordability of the robots is one key issue that has been widely flagged as a concern by multilevel stakeholders [13-15]. For instance, the JustoCat costs approximately US $1350 and PARO costs about US $6000. The substantial cost of such technology can reduce innovation dissemination [16], posing the ethical concern of unequal access [17]. Therefore, there is a need to explore lower costed alternatives.

**Figure 1:**
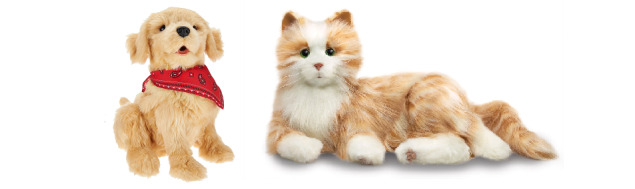
Joy for All robotic pets.

**Figure 2:**
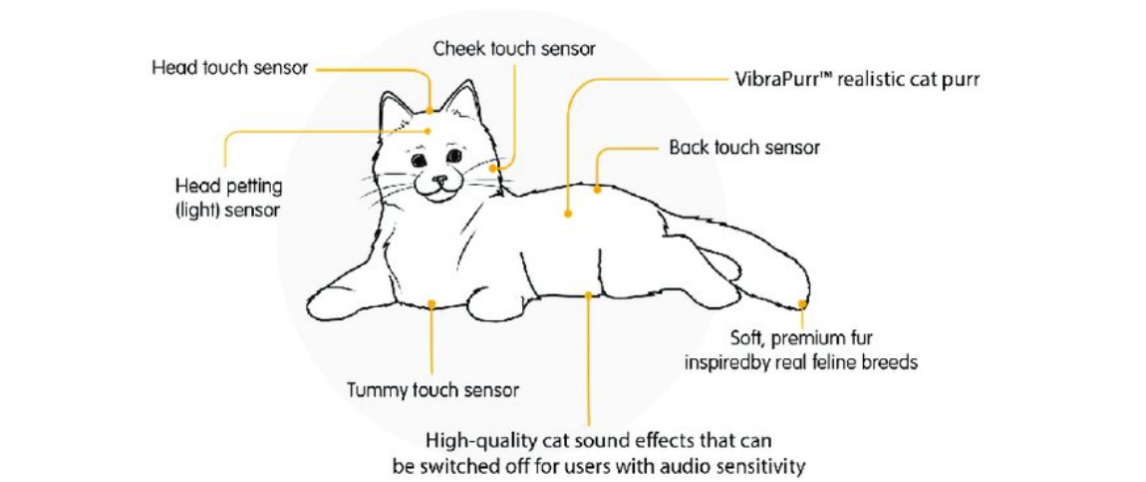
Touch interaction capabilities of the Joy for All cat. Used with permission from Joy for All.

The Joy for All (JfA) robotic pets have been identified as low-cost and commercially available innovations that have been used for older people and people with dementia [18]. They contain sensors to respond to touch and light, through movements and vocalizations, with the purpose of providing social interaction (Figures 1 and 2). Because they are capable of autonomous responses to stimuli for the purposes of social interaction, they should be considered as social robots. As one unit of the JfA robotic pet costs between US $110 and US $130, they are significantly more affordable. Synthesized findings from a recent review showed that despite being less-technologically advanced than other robotic pets, the JfA robotic pets showed promising benefits to address the psychosocial needs of older adults and people with dementia [18]. This included improved mood and affect, improved social interaction, companionship, and other well-being outcomes [18]. The lower cost of the technology also appeared to influence the ways in which the robotic pets were being used. For example, in contrast to other higher-costed pet robots that have been shared among users [12], most older adults and people with dementia that were included in the study owned their own JfA pet [18]. This implied that the affordability of the JfA pets had an influence on the accessibility to and adoption of this technology. Furthermore, individual ownership of social robots was suggested as a way to mediate the issue of infection control by reducing the potential for transmissible diseases from shared use. This is especially relevant in residential care settings in light of the COVID-19 pandemic, where a recent study has advised against the sharing of pet robots [19]. The review also found that while a few studies used both the JfA cat and dog for older adults and people with dementia, most only used the JfA robotic cat. A study by Bradwell et al [20] presented similar findings, where the JfA robotic cat, among 7 other alternatives, was chosen by older adults as their most preferred robotic pet.

Despite its potential as a therapeutic device, there is a lack of research to understand the usability and impact of the JfA cat based on perceptions and experiences of its use with older adults and people with dementia. As such, this study aims to explore the perceptions and experiences of using the JfA cat for older adults and people with dementia, using user-generated content published on consumer websites. This is a novel methodology that will be described below.

## Methods

### Study Data

The data used for this research are located on public platforms (ie, consumer review sites). Therefore, informed consent for this study was not obtained. However, as the use of direct quotes from consumer reviews could potentially make them identifiable, the quotes that were illustrated in this study were minimally amended to ensure users’ anonymity. This study was approved by the National University of Ireland Galway Research Ethics Committee (reference number R20.JUN.12).

### Focus on User-Led Content

To date, most research that aims to understand experiences using social robots has traditionally been researcher driven [10]. By contrast, this study utilized the large volume of information uploaded by users of the JfA cat onto publicly accessible online consumer review sites. These sites contain a sizeable body of anecdotal evidence from users who have purchased and used lower costed pet robots. These individuals shared detailed accounts of their experiences, for the primary benefit of other potential users who might be seeking to gather information about the product. Examining this valuable source of information during the study was an opportunity to develop knowledge shifting away from regarding researchers and health care professionals as the sole producers of information toward eliciting the voice and empowerment of nonprofessionals [21]. This approach has been used in other research fields, such as business or consumer research, however it is a novel methodology in the field of health and social sciences which allowed for an examination of user-led content.

### Data Collection: Data Sources and Search Strategy

Data collection involved 3 key steps. First, online consumer review sites were identified through a Google search, using the search terms “Joy for All cat” and “user review”. The researcher’s (WK) internet browsing history and cookies were cleared, and the search was conducted in the incognito mode. Next, the first 100 consumer sites identified from the Google search that contained consumer reviews of the robotic cat were selected as data collection sites. All reviews were manually extracted into Microsoft Excel. This step was essential to ensure a clear audit trail, as the content of a webpage may change depending on what the researcher searches for and researcher’s location [21]. Consumer reviews of all languages that were submitted up to July 24, 2020, were extracted using a standardized data extraction form (Multimedia Appendix 1) containing the following data fields: (1) review title, (2) review text, (3) star rating given, and (4) review date. Demographic information about users of the technology, such as their age group, diagnoses, and setting, was also collected if these data were available. If these were not available, the data field was left empty. To ensure anonymity, no potentially identifying information, such as the reviewing authors’ name and photo attachments, was collected. Finally, all reviews were screened to identify the sampling frame for data analysis.

### Inclusion and Exclusion Criteria

Reviews were included if they contained information about the use of the robotic cat for older adults or people with dementia in any settings and were published in the English language.

As not all reviews contained information regarding users’ age and diagnoses, innovative approaches had to be undertaken to ensure that all relevant reviews were adequately considered for inclusion. First, as the average age of becoming a grandparent is between 50 and 69 years in several countries [22-24], it seemed reasonable for the researcher to include reviews that mentioned about the use of the robotic cat for this group (ie, grandparents) as older adults. Next, reviews that contained information about the use of JfA cat in institutional care were also included, as the large majority of people living in assisted living facilities or care homes are of an older age group [25-29]. Hence, reviews that met any of the following inclusion criteria were included in the sampling frame:

- Included terms related to older adults, such as “older adult”, “elderly”, “elder”, “senior”, “grandmother”, or “grandfather” or explicit comment that users of the JfA robotic cat are aged 60 years and above
- Contained terms related to dementia, such as “dementia”, “Alzheimer’s disease”, “memory loss”, “memory problems”, “cognitive impairments” or “cognitive issues”, “memory care”
- Contained terms related to institutional care, such as “nursing home”, “assisted living facility”, “retirement home”
- Published in English language

All reviews that did not meet these inclusion criteria were excluded. Reviews that were included were cleaned and formatted on Microsoft Excel before being exported into NVivo 12 (QSR International) for data analysis.

### Data Analysis

Descriptive statistics was applied to characterize the number of reviews, available demographic information about users of the JfA cat, and the average star ratings given by users. Textual data were qualitatively analyzed using inductive content analysis, as described by Hsieh and Shannon [30], on the NVivo12 software. This method of data analysis was chosen as it guides systematic categorization of large volumes of text-based data and facilitates the identification of patterns of occurrences [31].

The data analysis proceeded as follows: First, 3 coders (WK, SW, and PH) immersed themselves in the data by reading all data repeatedly to obtain a sense of the whole and to allow new insights to emerge [31,32]. The first 5% of reviews were read word by word by each coder, who independently generated key thoughts or concepts for each phrase, and labeled them using descriptive and low-inference codes [33,34]. After that, all coders met to discuss similarities and differences, and agreed on codes that formed the initial coding scheme [30]. Next, this coding scheme was tested by WK, SW, and PH, who independently coded another 10% (n=137) of all data using the coding scheme. Data that did not fit into an existing code were assigned a new code. After this, intercoder reliability test (ICR), using the kappa coefficient (κ), was conducted to assess the similarity between the coding produced by the authors. Although there is no set consensus on what proportion of data should be analyzed to yield a reliable estimate of ICR [35], an analysis of 10%-25% of the data set is typical [36]. Conducting this test allowed the rigor and transparency of the coding framework to be ascertained [36-38]. The kappa coefficient of 0.60 was obtained, which demonstrated substantial agreement between coders [39]. Following this, all coders met to discuss and agree upon the final coding framework. In particular, they ensured that all data within the codes and categories were distinctive and that they had good coherence [40,41]. The final coding scheme (Multimedia Appendix 2) was tested by WK and SW, who independently coded another 5% (n=66) of the data set. Strong intercoder reliability was established (κ=0.7). Thereafter, the coding framework was applied to the remaining reviews by WK. Research rigor was ensured through prolonged engagement with the data [42], and frequent meetings with all coders throughout the creation of the coding framework, and to develop and refine the codes and categories.

## Results

### Overview

**Figure 3:**
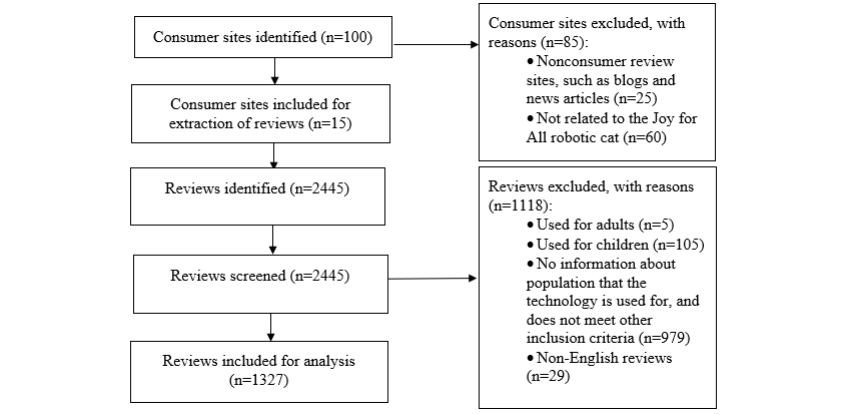
Flowchart (identification of reviews).

Figure 3 shows the flowchart that reports the data identification and collection. A total of 100 websites were identified, of which 15 were consumer review sites for the JfA robotic cat (Table 1).

**Table 1:** Consumer sites and reviews identified.

Consumer review sites (source)	Number of reviews
Amazon (total: 6 sites)	2068
Joy For All	214
Best Buy	25
MindCare Store	7
Eugeria	5
Caregiver Products	5
Alzstore	32
Alzproducts	10
QVC	79
Walmart	0

### Description of Reviews

A total of 2445 consumer reviews were submitted over a 5.5-year period from December 4, 2015, to July 24, 2020. Of these, 1327 reviews met the inclusion criteria and were included for data analysis. Most reviews were derived from consumer sites from the United States (n=948), Canada (n=132), the United Kingdom (n=80), and Australia (n=13). Most reviews contained information about review date and star rating (n=1309). Overall, the number of reviews increased steadily from 2015 to 2020, and its average star rating was 4.75 (Table 2).

**Table 2:** Star rating and number of reviews across the years.

Year of review	2015	2016	2017	2018	2019	2020
Number of reviews	15	180	222	228	372	292
Average star rating	4.13	4.63	4.86	4.74	4.76	4.76

### Review Authors and Users of the Robotic Cat

Information about the review authors and users is presented in Table 3. Most review authors were family members of the primary users of the JfA cat. The majority were children (n=770), grandchildren (n=120), and partners (n=52) of older adults or people with dementia. Only 2% (n=22) of all reviewers identified themselves as users of the robotic cat. Information about the relation of other review authors with the older person or person with dementia was not available in 247 (18.61%) cases.

**Table 3:** Information about review authors and users.

Information						Sample size, n (%) (N=1327)
**Review authors**						
	**Relationship to users**					
		**Family members**		1038 (78.22)		
			Children	770 (58.03)		
			Grandchildren	120 (9.04)		
			Partners	52 (3.92)		
			Other relatives	96 (7.23)		
	Self				22 (1.66)	
	Others (friends, care workers)				6 (0.45)	
	No information				247 (18.61)	
**Users**						
	**Age/diagnosis**					
		Older adults		586 (44.16)		
		People with dementia, cognitive impairment or memory issues		687 (51.77)		
	**Gender**					
		Female		988 (74.45)		
		Male		121 (9.12)		
		No information		218 (16.43)		
	**Setting**					
		Long-term care facilities		399 (30.07)		
		Memory care facilities		56 (4.22)		
		Retirement homes		16 (1.21)		
		Other care facilities		49 (3.69)		
		Own homes		19 (1.43)		
		No information		788 (59.38)		

The JfA cat was described as being for the use for older adults in 44.16% (586/1327) of reviews, while 51.77% (687/1327) described their use for people with dementia, cognitive impairment, or memory issues. The majority (n=1109) contained information about users’ gender, of which 89.09% (n=988) were females. Less than half (n=539) provided explicit information about the setting in which the device was used (Multimedia Appendix 3). Most were used in care settings, including long-term care facilities (n=399), specialized memory care facilities (n=56), retirement homes (n=16), or other care facilities (n=49).

### Qualitative Findings

#### Themes

Five themes were generated from the qualitative analysis: (1) prior expectations, (2) evolving perceptions, (3) meaningful activities, (4) impact of the robotic cat, and (5) practical aspects surrounding the use of the JfA cat. Table 4 shows the main themes, subthemes, and their prevalence in the data. It also provides information on exemplar codes and representative quotes in each subthemes. We will describe the themes in the following sections.

**Table 4:** Main themes, subthemes, and exemplar codes.

Main themes and subthemes			Prevalence, n (%)^a^		Examples of exemplar quotes *(code)*
**Prior expectations**					
	Circumstances	390 (29.39)		*When my 89-year-old mother was sent to a nursing home after a hospital stay, she lost her residence of 25 years, and worst of all, she lost her beloved orange tabby* *(can’t have a live cat).*	
	Expectations	182 (13.72)		*I was sceptical when I first heard that a mechanical cat like this could provide comfort and relief from anxiety for an elderly person suffering dementia (uncertainty and scepticism).*	
**Perceptions**					
	Appearance	364 (27.43)		*You can feel the bumps on the body through the fur (not lifelike).*	
	Interactivity	418 (31.50)		*It’s ingeniously designed, with the movements coming at a seemingly random cycle, just like a real animal. The meowing is the only weakness, it doesn't really sound like a cat, but the purring is spot on (positive comment about interactivity).*	
	Expectations met	415 (31.27)		*It did way more than I thought it could. Seemed like I found new things it could do for 3 days before I found everything (exceeded expectations).*	
	Ambivalence or rejection	114 (8.59)		*I bought this for my grandma, and she was very upset by it. She's in her late 80's and has slight dementia but she still got offended by this kitty. I took the cat home with me since she was so upset. I wasn't trying to insult her (rejection).*	
**Meaningful activities**					
	Companionship	270 (20.35)		*Now Brutus (name for the JfA cat) is helping my grandma not to feel completely alone (companionship).*	
	Doing something (activities)	500 (37.68)		*She takes it everywhere she goes, it rides along in her basket in her walker (taking it to places).*	
	Facilitation and support	75 (5.65)		*She wants it to purr, but gets upset if it meows too much. So we put it on mute so it still moves it’s head and eyes and arm and purrs but doesn’t get annoying (facilitation and support).*	
	Treating the robot cat as if it were real	70 (5.28)		*We talked to Mom/Grandma and let her know we were going to try to get her cat fixed. She is very concerned that we are going to take her cat away, but we assured her that we would try very hard to not take it away from her (attachment).*	
	Topic of conversation	78 (5.88)		*Both cat and grandfather are now quite popular. With dementia, I am not sure if he knows the cat is not real. Needless to say this cat has helped to improve my grandfather’s social interactions as many people come to check out the cat (topic of conversation).*	
**Impacts**					
	Positive impacts on users	1000 (75.36)		*Mom who has dementia & suffers from sundowner syndrome. Her cat’s meowing & purring (an impressively large repertoire of vocalizations) and the many movements it makes in response to touch, motion & sound provide the perfect kind of distraction my Mom needs in those PM hours (a welcome distraction).*	
	Negative impacts (users)	20 (1.51)		*She cried the other day because she thought it died (someone turned it off), she picked it up and cried for hours (negative impact on users).*	
	Positive impacts (others)	111 (8.36)		*My Mum is in a residential care manor and one of the other residents saw the cat and her daughter bought her one. All the residents love them (positive impacts on others).*	
	Negative impacts on others/caregivers	3 (0.23)		*When the care home residents saw the cat, there was a near riot because they all wanted to hold it and stroke it at the same time (negative impact on others).*	
**Practicalities**					
	Positive aspects	409 (30.82)		*We have had it for a few weeks now and have yet to replace the batteries. The cat goes into sleep mode when it is not touched for several minutes which saves the battery life. It is reactivated as soon as one of the sensors in the back or head are touched (battery life).*	
	Negative aspects	118 (8.89)		*The product is ONE STAR in terms of reliability. My FIL loved it so much he broke it. We think he held the head too tightly, and ultimately the servos broke. The cat still meows and purrs, but it no longer rolls onto its back and the eyes no longer open (not robust).*	
	Suggestions for improvement	51 (3.84)		*One thing they missed though, is the movement a cat makes when you scratch under her chin...You know, head back so you can really get in there. And if they are reading this...they could make it a smart cat with an app and everything. It would be cool if you could talk to it or give it commands and it responds (suggestions for improvement).*	

^a^Based on a total of 1327 reviews.

##### Prior Expectations

This theme describes the circumstances which prompted reviewers to acquire the JfA robotic cat for the older person or person with dementia, and reviewers’ perceptions of this technology prior to its use. Some reviewers (223/1327, 16.80%) commented that users had previous experience with or liked cats or other animals. However, users were now unable to own a live animal due to circumstantial or personal reasons (181/1327, 13.64%), such as institutional restrictions in residential care facilities and reduced physical or cognitive capacities.

Recently my 93 mother's dementia progressed to the point that she required assisted living in a nursing home. She was devastated that she could not take her two cats with her. She misses them more than anything.

Reviewer 108

Other reviewers indicated that they were prompted to purchase the JfA cat due to concerns about loneliness and isolation (102/1327, 7.69%), especially for intended users who lived alone or in residential facilities. The impact of COVID-19 measures was discussed in more recent reviews, where reviewers shared that visitation and activity restrictions exacerbated feelings of isolation. As such, expectations were focused on the users’ likes of animals, and hopes that it might provide comfort, companionship, and improve their overall quality of life.

When my family was faced with having to admit my 91-year-old Granny to a memory care facility it was devastating for us to think of her in there all alone and sad....

Reviewer 8

Due to the pandemic and imposed isolation and restrictions, all enrichment activities such as visiting music, games, exercises, therapy animals were ceased. Residents were no longer allowed to eat with other residents. We hoped the therapy cat would provide some comfort.

Reviewer 13

A few reviewers (70/1327, 5.28%) reported skepticism about the usefulness of the robotic pet, and concerns about how users would perceive it or respond to it.

I braced myself for a dismissive laugh, a ‘what the hell did you get this for, what a waste of money’.

Reviewer 335

At first, I was hesitant because I was worried that she (my mother) would be insulted if I gave her a ‘toy’.

Reviewer 146

##### Perceptions

This theme describes perceptions about the appearance and interactive features of the JfA cat, and whether it has met reviewers’ expectations. Perceptions about its appearance were mainly positive (312/1327, 23.51%), as reviewers commented about its life-likeness, size, and weight as resembling a real cat. Reviewers (357/1327, 26.90%) also commented about the device’s realistic movements and vocalizations, especially its purring. Some pointed out that their JfA cat looked similar to users’ previous cats. The robotic cat has sensors to respond to light and touch, however, its vocal and movement responses are nonprogrammable and are unpredictable. Some reviewers perceived its unpredictability as behaviors that resembled a live cat.

At intervals, this cat flicks its ears, raises a paw to its face as if it's washing, turns it head when touched, blinks its eyes, and partially closes its eyes; and purrs and meows when it's head and back are petted. It also rolls back to expose its belly, and what is funny about the cat, is that the moments are unpredictable, and spontaneous just as if it were real.

Reviewer 394

However, a few reviewers were negative in their comments (105/1327, 7.91%). The robotic cat was thought to be hard to the touch, which reduced its cuddliness and realism. The meowing sound of the cat was perceived as sounding like a person imitating its meow, and some movements were perceived to be mechanical looking and sounding. Although most reviewers said that not being life-like did not influence the interaction that users had with the technology, others commented that users’ acceptance of the device was negatively impacted.

She (my mother) doesn’t seem to notice the battery pack which is quite hard but likes to pet it (JfA cat) and keeps it on her bed at night.

Reviewer 588

The facial and ear movements do make some mechanical noise, but they're not that loud and don't detract from it. The one thing that I could do without is that occasionally the front half twists and rolls back, then after a few minutes it comes back up. That's when you hear the loud motor really kick in and I find it to be an unnatural movement.

Reviewer 215

While she (my mother) seemed to like the cat at first, she noticed the jerky movements and mechanical sounds it makes when it turns its head and she didn't like this. Three weeks after giving it to her she says that it's a beautiful cat, but that there's something wrong with it.

Reviewer 262

Perceptions of the JfA cat sometimes evolved with its use. Although most reviewers who discussed about their expectations of the robotic cat perceived it to have met or exceeded their prior expectations and fitted the needs of users (182/1327, 13.72%), some considered that the JfA cat may not be suitable for everyone. Similarly, a few users were ambivalent or had negative perceptions, and rejected the technology (72/1327, 5.43%).

We didn't know if (my father) would like it, scoff at it, or soon get bored with it. His eyes lit up the moment it (JfA cat) was taken out of the box.

Reviewer 171

My elderly aunt found the cat “creepy” and wanted no part of it. I can see how some elderly people would like this mechanical replica, but she didn't like it.

Reviewer 161

##### Meaningful Activities

This theme describes the engagement in meaningful activities with the JfA cat. Use of the JfA cat provided opportunities to supervise or provide care for older people and people with dementia (500/1327, 37.68%). Activities included holding, petting or brushing it, talking to it, keeping it on their laps, sleeping with it, and taking it to places. Some activities, such as naming the cat after their previous pet or loved ones, also provided an avenue for users to reminisce about past experiences. The robot’s interactivity also appeared to be perceived as behaviors of reciprocity, which facilitated users to continue engaging with it.

She (my mother) no longer speaks and appears somewhat catatonic. We were looking for ways to 'reach' her since talking to her and trying other activities were fruitless. We gave her this cat and got a glimpse into our mom again! The purring, meowing and movements awakened my mom and she came alive.

Reviewer 763

He (my dad) stroked her head, tail and back. He wanted to know her name. We told him she needed him to pick one for her. She became Fluffy! She meowed...He meowed back and laughed....

Reviewer 167

In some instances, the JfA cat was perceived to replace a lack of activity or participation, or replace undesirable or restless behaviors. Reviewers also commented that it provided companionship, and some users developed an attachment toward it.

She (my mother) has stopped looking for her kids at night and she is focused on taking care of her cat.

Reviewer 1060

She (my mother) will hang onto it (JfA cat) for dear life and not want to give it back to us. She has it with her at all times except at meals and during structured activities.

Reviewer 763

The JfA cat also provided users with a topic of conversation with others, including family members, friends, care providers, and residents within care facilities. Some passers-by would stop to interact with the user, talking about the JfA cat. This suggests that the robotic pet provided different opportunities for interactions.

She (my mother) had great difficulty speaking but would ask for “baby” every morning, would meow back at the cat and carry on an indecipherable conversation everyday.

Reviewer 641

I was delighted that not only did she (my mother) find it wonderful, but she also had the experience that all the dementia patients in her facility, including the nurses, are doting and cooing at the kitty cat. I was pleased that it brought her comfort and joy from the attention she got as well as the kitty itself.

Reviewer 651

Users varied as to whether they considered the JfA cat to be real. Reviewers (74/1327, 5.58%) mentioned that users were aware that this was not a live cat, but still enjoyed the device. While some commented about explicit attempts to introduce or remind users that the JfA cat is a robotic device, others suggested that users should treat it as a real cat. Some users who were not aware that the JfA cat was a robotic device treated it as if it were a live animal (70/1327, 5.28%) and tried to feed it with food and water, which dirtied it. Such perceptions also caused anxiety among some users, who became concerned that it would not eat or drink, or that it would escape. The device’s vocalizations caused concerns among some users (70/1327, 5.28%), who became worried that the cat was upset. Some also exhibited distress when the robotic cat was not moving.

It’s unclear whether she (my mother) believes it (JfA cat) is real or not - but we avoid clarifying that it isn't, and all try to act interact with it in front of her as though it is real, and of course we helped her pick a name!

Reviewer 594

Dad was nervous his cat would escape and get lost or that no one had given her food or water and she'd die. Mom had to stop him from bring Fluffy water (i.e., dumping it over her).

Reviewer 167

##### Impact of the Robotic Cat

This theme describes how the JfA robotic cat impacted the primary user and the caregiver. Most reviewers (874/1327, 65.86%) reported that users exhibited positive emotions. These included expressions of love and affection toward the robotic cat, expressions of joy, and improved mood. Several reviewers (228/1327, 17.18%) also commented that use of this technology was calming, provided comfort, and gave users a sense of purpose.

She [my mother] now has a reason to get out of bed in the morning and is back to her old self again.

Reviewer 554

I would say this week has been his calmest, happiest, most relaxed, enjoyable week in possibly three or more years! Because of this life-like, mechanical companion designed exactly for people like him.

Reviewer 167

She never slept through the night. Usually, I am up with her constantly, but we actually had to wake her this morning. She actually went to sleep with her cat cuddled in her arms.

Reviewer 160

The reviewers and other caregivers were also impacted. Reviewers shared about positive emotions and physical relief that they, their family members, and care staff experienced from observing users’ interactions with the robotic cat (161/1327, 12.13%). Amidst these feelings, some reviewers shared about a sense of conflict or dilemma in watching users interact with a robotic device.

The amount of joy this has brought her - and me watching her interact with the cat - is priceless.

Reviewer 265

Now honestly for some in my family the idea that my mom is in love with a mechanical cat and believes it is real can be a distressing and shocking new reality. But to see her joy with this cat and to occasionally use it as a diversion when she sundowns or when she goes through an angry phase is priceless.

Reviewer 530

The JfA cat was also reported to have a positive impact on other people (111/1327, 8.36%), such as users’ neighbors, or other residents in their care facility, who also enjoyed the technology.

She enjoys sharing it with all the other residents, and they agree that petting this purring cat is very soothing and relaxing.

Reviewer 146

##### Practical Aspects of Its Use

This theme describes comments about the facilitation that was rendered to support users’ interaction with the JfA cat, overall experiences of the technology, and technical aspects of its use. Some reviewers provided mediation and supported users who perceived it to be a real animal (75/1327, 5.65%). Actions included reassuring users that the JfA cat was well taken care of, keeping it on mute or turning it off at night when users fell asleep, preparing spare batteries and being ready to prepare to change them as needed, and regularly cleaning food stains off its mouth. A few mentioned the use of a waterproof bib on the JfA cat’s neck, and creating artificial feeding stations. Some reviewers also commented that they purchased an additional robotic cat as a back-up device.

It was purring a lot last night and I heard him telling the cat “shhhhh”. I looked over and he's looking it in the eyes and shhhhing it. So I turned the cat off for a while.

Reviewer 722

I've got her (JfA cat) a collar and made her a tag and a feeding station (thank you hot glue and modge podge), so that he can care for her the way years of instinct and memories tell him he should.

Reviewer 167

Overall, most reviewers (409/1327, 30.82%) reported positive experiences. This included comments about satisfaction, and comments that they would recommend this device to others.

If you have someone in your life living with dementia or Alzheimer's, or something similar, please consider...this for that person. I haven't seen my grandmother that happy since before she became sick.

Reviewer 180

Nevertheless, some reviewers (118/1327, 8.89%) shared negative experiences, which included comments about the technical aspects of its use. Experiences about the JfA cat’s technical performance were mixed. While some reviewers shared that the technology was durable and lasted for over a year at the time of review (32/1327, 2.41%), others commented that it only lasted for a week to 8 months (48/1327, 3.62%). Others elaborated that the short lifespan of the device was sometimes attributed to users’ behaviors, such as attempts to feed it or holding it too tightly, which hindered or damaged the device’s mechanics. Such issues led to disappointment among some reviewers.

Grandma holds it so tight that when the cat wants to put its paw up or roll on its back, she is preventing the movement. Now, it sounds like the motor has been damaged.

Reviewer 344

It’s really sad that this cat did not last. My elderly mother is devastated....Really, really, really disappointed.

Reviewer 207

Some reviewers also raised concerns about difficulties cleaning the robotic cat and maintaining its cleanliness.

Ours is showing wear around the cat’s mouth as grandma keeps insisting on feeding it real food...so I am cleaning it ALOT with dove soap, water and a washcloth.

Reviewer 265

It is difficult to clean Lucette's (name for the JfA cat) fur. Elderly people do tend to be like children and stroke their pets with sticky hands.

Reviewer 108

Finally, some reviewers (51/1327, 3.84%) suggested how the JfA cat could be improved. These included improvements to its appearance, such as having more cushioning to make it softer to hold, having a more realistic “meowing” sound, and more interactive movements. Reviewers also commented that the device should be more durable and customizable, and suggested that volume controls or options to turn off the movement of the cat while keeping its sounds on should be made available.

## Discussion

### Principal Findings

This is the first study to use a novel web-based approach to explore the usability and impact of a low-cost robotic pet for older adults and people with dementia, based on perceptions and experiences of its use. Most of the review content was derived from consumer sites that were based in the United States, and most reviewers were family members of older adults and people with dementia. Overall, most reviewers had positive perceptions and experiences of using the JfA cat and found it to be beneficial and practical for older adults and people with dementia. Nevertheless, not all were satisfied with this technology.

Users’ previous experiences of pet ownership were frequently reported as a circumstantial reason for purchasing the JfA cat for the intended user. This finding aligns with previous findings that users’ like of animals influenced their acceptance of a robotic pet [43]. Therefore, it may be worth screening users’ likes and dislikes of animals as a predictor to gauge their acceptance of the robotic pets [44]. Reviewers also acknowledged that pragmatic deterrents, such as institutional regulations and a lack of capacity to care for a live animal, propelled them to seek robotic alternatives. This echoes the proposition that a recognition of the relative advantage of an innovation can facilitate its adoption [45].

Most perceptions about the JfA cat were positive, which suggests its design as a familiar animal was acceptable. In previous studies, familiarly designed robotic animals, such as the JustoCat and the NeCoRo cat, were also well received by older adults and people with dementia [46,47]. Likewise, other studies have highlighted preferences for familiarly designed pet robots [20,48,49]. These findings contrast with the notion that people are more likely to accept less familiarly designed robots because they would have fewer prior conceptions or expectations [50]. However, this hypothesis has not been widely evaluated, as few studies have investigated design preferences of older adults or people with dementia. Indeed, in most research studies, participants were typically given a single pet robot to engage with, which was selected based on the needs of the research rather than the preference of the participants. In line with a person-centered approach to care [51], older adults and people with dementia should be given the autonomy to choose their preferred robotic pet design. People with dementia, especially in the advanced disease stages, may not be able to articulate their preferences for pet design. However, they should still be given opportunities to participate in decisions relating to their care [52], to allow for the maintenance of self-identity, dignity [53], and personhood [54]. Moving forward, more considerations should be made to identify pet robot design preferences of individuals.

Use of the robotic cat offered older adults and people with dementia opportunities to participate in meaningful activities. Older adults and people with dementia participated in an array of activities with the JfA cat, such as talking to it and about it, cuddling, and stroking it. These findings resonate with results from studies which used other robotic pets [46,48,55,56], suggesting the potential of the JfA cat to elicit similar activities. Other activities identified included brushing the cat, sleeping with it, and taking it to places. Some reviewers supported these meaningful activities by getting a brush for users to brush the cat, and getting a cat bed and a personalized collar to allow for easier identification in care facilities. Such activities were not reported in previous studies and appeared to be unique to this study. This might be attributed to more opportunities for interaction with the cat over an extended period, made possible due to individuals owning their own robotic cat and not sharing it with others. Individual ownership may have provided users with the opportunity to take ownership of the robotic pet and be actively involved as care providers, in contrast to their traditional role as passive recipients of care [57]. Furthermore, the consistent and proximate presence of the JfA cat might have enabled such additional activities involving its use to be scaffolded naturally.

The relationship between engagement in meaningful activities and health outcomes has been established [58-62]. Similar to findings from previous studies [10-12,18], participating in activities with the JfA cat elicited positive emotions among users, and also provided comforting and calming effects. This is an important finding, because it highlights the potential of the JfA cat to elicit therapeutic benefits that are similar to costlier and more technologically sophisticated robotic pets. This raises an important question—In consideration of potential cost benefits, what degree of technological sophistication is required for a robotic pet to be therapeutic? Further research and randomized controlled trials should be conducted to evaluate and compare the effectiveness of low-cost robotic pets on the mental and social health of older adults and people with dementia, with other more technologically advanced robots.

The movements and vocalizations of the JfA cat appeared to be perceived positively by users as behaviors of reciprocity. Reciprocity, or the give and take that occurs between individuals, can influence the maintenance of social relationships [63,64]. This may explain why interactive robotic pets have been able to elicit more user engagement as compared with noninteractive or plush alternatives [65,66]. Interestingly, the lack of predictable responses to touch and movement was interpreted by some users as resemblant of a live cat’s behavior, and was well received. Nevertheless, the JfA cat’s interactive features also resulted in some negative impacts, particularly among those who perceived it as a live animal. When the robotic cat ran out of batteries, some users exhibited emotional distress as they perceived it to be dead. The meowing sounds worried or caused annoyance to some users, who sometimes perceived the robotic cat to have unmet needs. Similar issues have also been raised previously in relation to other robotic pets [13,48,67,68]. Furthermore, some users became concerned that the cat was not eating and attempted to feed it. These issues may be due to individual ownership of the robotic cat, where perceived responsibility for pet care may place a burden on people with cognitive impairment [69]. In such instances, reviewers provided mediation and support. This suggests that unattended, prolonged interactions with the robotic pet may have the potential to cause negative impacts. In turn, this raises the question as to what amount of robot–human interactions, especially for people with cognitive impairments, should be conducted completely without the support of caregivers. Findings from this study suggest some degree of facilitation and mediation by caregivers may still be necessary.

The JfA cat also positively impacted caregivers, providing them with a sense of relief and positive emotions, which included feelings of happiness and contentment. There is currently a lack of research that has focused on how robotic pets impact caregivers. More research is needed to increase understanding, especially since one of the key premises for developing social robots is to supplement and support the care of older people with dementia [66].

Finally, despite the overall positive perceptions and experiences, some reviewers reported negative opinions about the cat’s design. This included comments about its “hardness” and lack of sophistication, such as audible mechanics during movements and unrealistic “meowing” sounds. These issues did not appear to influence most users’ interaction with the robotic cat, suggesting that reviewers may have a higher expectation than the end users in wanting the robotic cat to behave more realistically and autonomously. Nevertheless, these issues resulted in the rejection of this technology by a minority of users. Comments about the robustness of the technology were mixed, with some reviewers being dissatisfied with its durability. Some elaborated that users’ handling of the JfA cat, such as holding it too tightly or dropping it, affected its functioning. The relatively short longevity of the device has potential to cause negative impacts such as emotional distress, especially among users who have developed an attachment toward it [70]. The understanding of such issues are useful to inform future robot development to ensure technological robustness [18].

### Limitations

Despite the valuable new knowledge that was generated through this study, there are limitations that should be acknowledged. Data that were used for this study were self-reported information that was gathered through publicly available sources. The anonymity of users makes it difficult to verify the authenticity of the content, and to verify the ages and diagnoses of the users of the robotic cat. Most reviewers were family members, and as such, their perceptions and experiences might differ from actual opinions of the primary end user (ie, older adults or people with dementia). Although most included reviews were shown as verified purchases, it is not possible to confirm the authenticity of review or distinguish potentially deceptive reviews. There could also be a bias in terms of the representation of data, as not all consumers will upload their reviews on consumer websites. Nevertheless, given the analysis of the large number of reviews from multiple websites across a 5-year period, as well as the richness of the data contained in these reviews, it may be reasonable to infer that the findings from this study represent real-world perceptions and experiences of using the JfA cat for older adults and people with dementia.

### Conclusion

This study provides important knowledge about the usability and impact of a low-cost robotic pet for older adults and people with dementia based on perceptions and experiences of its use. It analyzed user-driven content to access a unique perspective toward an understanding of this phenomenon. We found that circumstantial reasons, such as inability to care for a pet, have prompted the use of the robotic cat, and that familiarly designed robotic pets can be accepted by older adults and people with dementia. Although the JfA cat is less technologically advanced than other robotic pets, its interactive features were generally well received. Use of the JfA cat facilitated participation in meaningful occupations, as it provided older adults and people with dementia opportunities to participate in various activities. These activities elicited positive psychosocial impacts on both users and caregivers. Nevertheless, facilitation by caregivers may be necessary to monitor for and mitigate potential negative impacts. Although perceptions and experiences were mainly positive, negative aspects of the JfA cat’s design and interactivity were raised. Experiences of its durability were also mixed, which highlights the need to improve the technical robustness of this device.

These insights are vital in helping researchers, robot developers, and clinicians to understand the viability of using low-cost robotic pets to benefit older adults and people with dementia. Future research should consider evaluating design preferences for nonfamiliarly versus familiarly designed robotic pets. It will also be valuable to conduct a randomized controlled trial to compare the impacts of low-cost robotic pets with other more technologically advanced robotic pets, to understand any similarities or differences of their impacts on the mental and social health of older adults and people with dementia. A process evaluation may also be conducted to identify factors that may explain any outcome variations. This has the potential to influence equal access to technology if their impacts on the psychosocial health of users are comparable.

## Appendix

Multimedia Appendix 1 Data extraction form.

Multimedia Appendix 2 Coding framework.

Multimedia Appendix 3 Settings.

## Abbreviations

ICR: intercoder reliability test
JfA: Joy for All
