# Supplementary material for: The Usability and Impact of a Low-Cost Pet Robot for Older Adults and People With Dementia: Qualitative Content Analysis of User Experiences and Perceptions on Consumer Websites
Source: JMIR Aging. 2022 Feb 22;5(1):e29224. doi: 10.2196/29224 (PMC8905483; doi:10.2196/29224)
Supplement: Multimedia Appendix 2 [file aging_v5i1e29224_app2.docx]

# **Summary of all codes, sub-categories, and categories**

| 1. Prior expectations | Circumstances | Can’t have a real cat |
| --- | --- | --- |
|  |  | Covid-19 |
|  |  | Isolation |
|  |  | Likes cats |
|  | Expectations | Advantages over live cat |
|  |  | Improve QoL |
|  |  | Uncertainty and ambivalence |
| 1. Perceptions | Appearance | Looks real |
|  |  | Not real |
|  | Interactivity | Negative comments about interactivity |
|  |  | Positive comments about interactivity |
|  | Expectations met | Exceeded expectations |
|  |  | Gratitude |
|  |  | Ideally fitting needs |
|  | Not fitting needs | - |
|  | Awareness that the cat is a robot | Aware that the cat is not real |
|  |  | Not aware that the cat is not real |
|  |  | Occasionally aware |
|  | Ambivalence and rejection (Primary users) | Confusion about the cat |
|  |  | Negative reactions |
|  |  | Tepid responses |
|  | Ambivalence and rejection (Primary users) | Conflicted or tepid responses |
|  |  | Negative responses |
| 1. A meaningful occupation | Attachment to the cat |  |
|  | Companionship | - |
|  | Doing something with the cat | Brushing |
|  |  | Holding, stroking, or patting |
|  |  | Keep it on lap |
|  |  | Naming the cat |
|  |  | Replaces other activities/lack of activities |
|  |  | Sleeping with the cat |
|  |  | Taking the cat to places |
|  |  | Talking to the cat |
|  | Facilitation and support | - |
|  | Reminiscence | - |
|  | Treating it as if it is real | - |
|  | Shows off cat to others | - |
|  | Topic of conversation | - |
| 1. impacts | Positive impacts on primary user | A welcome distraction |
|  |  | Comforting and calming |
|  |  | Positive emotions |
|  |  | Sustained effects |
|  | Positive impacts on caregivers | Positive emotions |
|  |  | Caregiver relief |
|  | Positive impacts on others | Positive emotions |
|  |  | Sharing the pet |
|  | Negative impacts on primary user |  |
|  | Negative impacts on secondary user or others |  |
| 1. Practicalities | Expensive | **-** |
|  | Negative aspects | Battery |
|  |  | Hygiene |
|  |  | Not robust |
|  |  | Not worth the money |
|  |  | Technical malfunction |
|  |  | Volume |
|  |  | Disappointment |
|  |  | Will not repurchase |
|  | Positive aspects | Battery |
|  |  | Robust |
|  |  | Volume |
|  |  | Recommend to others |
|  |  | Satisfied with purchase |
|  | Suggestions for improvement | - |

# **Detailed description of codes, sub-categories and categories**

| **Category** | **Subcategory** | **Code** | **Explanation** |
| --- | --- | --- | --- |
| Prior expectations Prior expectations of the robotic cat by users before actual use. This can include their perceptions of who the robotic cat should be used for, when it should be used for and what they hope for it to do. | Circumstances Personal/environmental circumstances which influenced users' perceptions of the potential value of the robotic cat.   - Personal circumstances e.g. loneliness, past experiences with cats/animal lover. - Environmental circumstances e.g. unable to have a live animal | Can’t have a real cat | not able to have a real (live) cat or animal due to circumstantial reasons (e.g. not allowed to) or personal reasons (e.g. inability to care for a real animal) |
|  |  | Covid-19 | Influence of COVID-19 pandemic on their perceived utility of the robotic cat (i.e., did they purchase the cat because they perceive it to have benefits to combat effects of the pandemic)? |
|  |  | Isolation | primary users (older person or person with dementia) are lonely or isolated |
|  |  | Likes cats | previously owned cats, or comments that the primary user (older person/PwD) like cats or have had cats. May also contain comments that users like plush toys/soft toys |
|  | Expectations Expectations of what the cat can offer to the primary user (i.e. older person or person with dementia) | Advantages over live cat | benefits of the robotic cat compared to having a live cat |
|  |  | Improve QoL | expectations/hopes to improve the quality of life (positive dimensions, e.g. comfort, joy etc) of the primary user (older person/person with dementia) |
|  |  | Uncertainty and ambivalence | uncertainty, ambivalence, or initial scepticism about how the robotic cat may impact the primary user (i.e. older person or person with dementia) |

| **Category** | **Subcategory** | **Code** | **Explanation** |
| --- | --- | --- | --- |
| Perceptions Initial perceptions and evolution of perceptions after using the robotic cat. This includes their perception of the appearance and interactivity of the cat (after seeing/using it), whether it meets their initial perceptions (and expectations), and reactions to these perceptions | Ambivalence and rejection  (Primary users) Ambivalence towards or rejection of the robotic cat by primary users | Confusion about the cat | confusion about the robotic cat's actions, purpose or intentions |
|  |  | Negative perceptions | negative perceptions towards the robotic cat or taking offense to being given the robotic cat. note that this should contain comments that are related to **perceptions.** (for comments about reactions to the cat, code it to one of the codes in the **“Impacts”** category instead). |
|  |  | Tepid perceptions | References to positive but tepid (somewhat neutral) responses |
|  | Ambivalence and rejection (Secondary users) Ambivalence towards or rejection of the robotic cat by secondary users | Conflicted or tepid perceptions | conflicted responses or feelings towards the robotic cat, or tepid responses. note that this should contain information about **perceptions.** (for comments about reactions to the cat, code it to one of the codes in the **“Impacts”** category) |
|  |  | Negative perceptions | explicit dislikes of the robotic cat as perceived by the caregiver (this should contain comments relating to their **perceptions.**  If there were negative impacts on secondary users, such as having stress as a result of using the cat, code it to “negative impacts on caregivers”. |
|  | Appearance Appearance (general outlook and design) of the robotic cat: e.g. fur covering, lifelikeness  *Note: for comments relating to functions of the cat, e.g. sounds or movement, they should be coded in "interactivity"* | Looks real | positive comments that the robotic cat looks real. this can include comments about the fur, size, feels of the robotic cat |
|  |  | Not real | Negative comments that the robotic cat looks unreal. this can include comments about the fur, size, feels of the robotic cat |
|  | Awareness that the cat is a robot Primary users’ awareness that the Joy for All cat is a robotic device and not a live animal | Aware that the cat is not real | primary user is aware that the robotic cat is real or not real (i.e. a robotic device) |
|  |  | Not aware that the cat is not real | primary user is **not** aware that the robotic cat is real or not real (i.e. a robotic device); or thinks that the robotic cat is a live animal |
|  |  | Occasionally aware | primary user is occasionally (or sometimes) aware that the robotic cat is not real |
|  | Interactivity Interactivity of the robotic cat. this includes sounds (meow, purring) or movement (turning head, rolling over).  *For any comments relating to the appearance/outlook/design of the cat, they should be coded to "appearance".* | Negative comments about interactivity | overall negative comments about the interactivity of the robotic cat. this includes sounds (meow, purring) or movement (turning head, rolling over) |
|  |  | Positive comments | overall positive comments about the interactivity of the robotic cat. this includes sounds (meow, purring) or movement (turning head, rolling over) |
|  | Expectations met Comments that the robotic cat met or exceeded users' expectations | Exceeded expectations | explicit expressions that use of the robotic cat exceeded the user's expectations |
|  |  | Gratitude | expressions of gratitude from having the robotic cat |
|  |  | Ideally fitting needs | perception that the robotic cat is ideal and suits users (older person or person with dementia)'s needs |
|  | Not fitting needs | - | (explicit comment) about the perception that the robotic cat is not ideal for the older person or person with dementia’s needs |

| **Category** | **Subcategory** | **Code** | **Explanation** |
| --- | --- | --- | --- |
| A meaningful occupation Describe the meaningfulness (i.e. utility) of the robotic cat to older persons/people with dementia as an occupation.  This can include how the robotic cat serves to provide a sense of meaning to the person (e.g. providing a meaningful occupation or promotes occupational engagement) | Attachment to the cat |  | negative thoughts and emotions of user due to dependence on cat and problems when it was not working or around the prospect of it being withdrawn. Indications of attachment and negative emotions when attachment interrupted. |
|  | Companionship | - | perceptions that the cat provided companionship |
|  | Doing something with the cat Things/activities that can be done with the cat, such as stroking or brushing it, naming it, keeping it on lap etc | Brushing | activity of brushing or combing the cat's fur |
|  |  | Holding, stroking, or patting | activity of cuddling (or holding), stroking or patting the cat |
|  | Companionship | Keep it on lap | activity of placing or keeping the robotic cat on primary users' laps |
|  | Doing something with the cat Things/activities that can be done with the cat, such as stroking or brushing it, naming it, keeping it on lap etc  Facilitation and support  Reminiscence  Rough or undesirable behaviours | Naming the cat | primary user's naming of the cat |
|  |  | Replaces other activities/lack of activities | References to person not having access to alternative activities |
|  |  | Sleeping with the cat | placing the cat by the cat or sleeping with the robotic cat in close proximity (deliberate intent) |
|  |  | Taking the cat to places | comments that primary user deliberately took the robotic cat to places where he/she went |
|  |  | Talking to the cat | comments that the primary user talked to the cat |
|  | Facilitation and support | - | statements that relate to any form of support that is provided to the older person to use the cat |
|  | Reminiscence | - | comments that the robotic cat provided the primary user with an opportunity to reminiscence about previous events/preferences |
|  | Treating it as if it were real | - | This encompasses any actions/behaviours that are not already mentioned in “doing something with the cat” – such as actions to suggest that they are being protective, or feeding the cat or otherwise looking after the cat)  *For any codes that can be coded* ***to “doing something with the cat” or any other codes in this category,*** *try not to code them again here, to avoid capturing of duplicate meanings.* |
|  | Shows off cat to others | - | expressions that the primary user deliberately shows off his/her robotic cat to others |
|  | Topic of conversation | - | the robotic cat is something that the primary user can talk about with family or caregivers, kind of a conversation starter |

| **Category** | **Subcategory** | **Code** | **Explanation** |
| --- | --- | --- | --- |
| Impacts Description about the positive and negative impacts of using the robotic cat | Positive impacts on primary user positive impacts of the robotic cat on the primary user (i.e. older person or person with dementia) | A welcome distraction | references to the cat being a distraction to user or a focus for them |
|  |  | Comforting and calming | comfort and calming effects that are derived as a use of the robotic cat. this code may also contain comments about reduction of negative behaviours (e.g. reduced anxiety, reduced stress) |
|  |  | Positive emotions | experiences of positive emotions associated with use of the robotic cat, such as joy, laughter, affection/love, enjoyment and smiles |
|  |  | Sustained effects | comments about the sustained effects (or longer term effects) of the robotic cat |
|  | Positive impacts on caregivers | Positive emotions | positive emotions that formal and informal caregivers experience as a result of direct or indirect interaction with the robotic cat |
|  |  | Caregiver relief | relieve caregivers' concerns |
|  | Positive impacts on others | Positive emotions | References to cat benefitting other people than the primary user who may or may not be caregivers, including their expressions of enjoying the cat (i.e. effects on the user's social environment) |
|  |  | Sharing the pet | comments that other people (apart from users and caregivers) take turns/share the use of the robotic cat |
|  | Negative impacts on primary users | - | negative emotions or other impacts that are derived from the use of the robotic cat. This code may contain comments about the **increase in negative behaviours** (e.g. increased anxiety, increased stress or agitation). If the code is related to perceptions, code it within in the “perceptions” category. |
|  | Negative impacts on caregivers or others | - | negative emotions that caregivers or other stakeholders experience as a result of direct or indirect interaction with the robotic cat |

| **Category** | **Subcategory** | **Code** | **Explanation** |
| --- | --- | --- | --- |
| Practicalities experiences relating to other practicalities involving the robotic cat, including its affordability, technical functions, and robustness. also includes users' overall satisfaction regarding their purchase of the robotic cat | Expensive | **-** | comments stating that the robotic cat is expensive or costly |
|  | Negative aspects negative aspects relating to the technical aspects and practical use of the robotic cat in the real world (e.g. hygiene, infection control), as well as overall dissatisfaction with the purchase  *(dissatisfaction about the specific functions of the cat should be coded elsewhere, e.g. appearance/interactivity - negative aspects)* | Battery | negative experiences relating to the battery life (or other battery related issues) of the robotic cat |
|  |  | Hygiene | negative experiences or concerns regarding the hygiene or infection control aspects of using the robotic cat |
|  |  | Not robust | poor quality or overall condition of the robotic cat |
|  |  | Not worth the money | expressions that the robotic pet is not worth purchasing (in relation to costs) |
|  |  | Technical malfunction | all other general technical issues that has resulted in errors or malfunction, or non-function of the robotic cat |
|  |  | Volume | negative comments relating to the volume of the robotic cat and volume related controls |
|  |  | Disappointment | general expressions that the user's negative experiences and disappointment in this purchase |
|  |  | Will not repurchase | comments that the user will not repurchase the robotic cat, or recommends against the purchase of the robotic cat |
|  | Positive aspects positive aspects relating to the technical aspects and practical use of the robotic cat in the real world, as well as overall satisfaction with the purchase.  (satisfaction about specific functions coded to “appearance”, or on "ideally fitting needs" if the comment is regarding how it fits the needs of users) | Battery | positive experiences relating to the battery life (or other battery related issues) of the robotic cat |
|  |  | Robust | comments about the good quality or overall condition of the robotic cat |
|  |  | Volume | positive comments relating to the volume of the robotic cat and volume related controls |
|  |  | Recommend to others | Comments that the user would recommend the robotic pet to other users with specific needs |
|  |  | Satisfied with purchase | overall satisfaction of purchase of the robotic cat |
|  | Suggestions for improvement | - | suggestions on how the cat may be improved, based on users' experiences |
