# Supplementary material for: The Usability and Impact of a Low-Cost Pet Robot for Older Adults and People With Dementia: Qualitative Content Analysis of User Experiences and Perceptions on Consumer Websites
Source: JMIR Aging. 2022 Feb 22;5(1):e29224. doi: 10.2196/29224 (PMC8905483; doi:10.2196/29224)
Supplement: Multimedia Appendix 3 [file aging_v5i1e29224_app3.docx]

**Multimedia Appendix 3: Setting**

| **Setting** | **Other terms used** | **No. of reviews** |
| --- | --- | --- |
| Long term care facility | Nursing home, Care home, aged care home, old age home, old folks home, personal care home, residential care home, residential care facility, residential care, residential care facility, assisted living, assisted living centre, long term care facility, senior assisted living, senior assisted home, senior assisted residence, independent living facility | 399 |
| Memory care facility | Memory care, memory support | 56 |
| Lives alone | - | 19 |
| Retirement home | Retirement community, retirement residence, retirement community | 16 |
| Other dementia care facilities | Dementia unit, dementia wing, dementia care community residence, dementia facility, Alzheimer's facility, dementia care community residence, dementia facility, Alzheimer's facility, special care dementia unit, Alzheimer’s day care, dementia drop in cent | 13 |
| Others | Hospital, rehab facility, nursing rehab facility, elderly care facility, sheltered housing, adult foster home, group home, hospice, skilled care facility | 36 |
